# Evaluation of *commotio retinae* in orbital fractures

**DOI:** 10.5935/0004-2749.2021-0456

**Published:** 2022-10-19

**Authors:** Yael Steinberg, Matthew S Wieder, Ksenia Denisova, Catherine He, Afshin Parsikia, Joyce N Mbekeani

**Affiliations:** 1 Medical School Training Program, Albert Einstein College of Medicine, Bronx, NY, USA; 2 Department of Ophthalmology, New York Eye & Ear Infirmary of Mount Sinai, New York, NY, USA; 3 Department of Ophthalmology & Visual Sciences, Montefore Medical Center, Bronx NY, USA; 4 Department of Ophthalmology, Yale School of Medicine, New Haven, CT, USA; 5 Research Services, University of Pennsylvania, Philadelphia, PA, USA; 6 Department of Surgery (Ophthalmology), Jacobi Medical Center, Bronx, NY, USA; 7 Department of Ophthalmology & Visual Sciences, Albert Einstein College of Medicine, Bronx, NY, USA

**Keywords:** Orbital fractures, Eye movements, Retina, Commotio and injuries, Fraturas orbitárias, Movimentos oculares, Retina, Ferimentos e lesões

## Abstract

**Purpose:**

This study aimed to evaluate the mechanisms of injury and types of orbital fractures and their relation to concurrent commotio retinae.

**Methods:**

This retrospective study evaluated the records of patients with orbital fractures whose diagnoses had been confirmed by computer tomography between July 2017 and September 2019. Patient demographics, the circumstances of injury, ophthalmic examination results, and radiological findings were tabulated. Statistical analysis of the data used two-tailed student’s t-tests, chi-squared tests, and odds ratio calculations. Statistical significance was set at p<0.05.

**Results:**

Of the 204 patients with orbital fractures included in this study, 154 (75.5%) were male. The mean age was 42.1 years. Orbital fractures involving one orbital wall (58.8%) were more common than those affecting multiple walls (41.2%). The majority of fractures affected the inferior wall (60.3%), with the medial walls being the next most frequently affected (19.6%). The most common cause of injury was assault (59.3%), and the second most common was falls (24%). Commotio retinae was observed in 20.1% of orbital fracture cases and was most associated with injuries caused by assault (OR=5.22, p<0.001) and least associated with those caused by falls (OR=0.06, p<0.001). Eye movement restrictions were more common in central than peripheral commotio (OR=3.79, p=0.015) and with medial wall fractures than fractures to other orbital walls (OR=7.16, p<0.001). The odds of commotio were not found to be higher in patients with multi-walled orbital fractures than in those with single-walled fractures (p=0.967).

**Conclusions:**

In the study population, assault was the most common cause of orbital fractures and resulted in commotio retinae than other causes. Ophthalmologists should be aware of the likelihood of commotio retinae in patients with orbital fractures resulting from assault, regardless of the extent of the patient’s injuries.

## Abstract (pt)

**Objetivo:**

Este estudo visou avaliar os mecanismos da lesão e os tipos de fraturas orbitárias e sua relação com *commotio retinae* simultânea.

**Métodos:**

Este estudo retrospectivo avaliou registros de pacientes com fraturas orbitárias cujos diagnósticos foram confirmados por tomografia computadorizada entre julho de 2017 e setembro de 2019. Foram registrados os dados demográficos, circunstâncias da lesão, os resultados do exame oftalmológico e achados radiológicos. A análise estatística dos dados usou os testes de *t-Student* bicaudal, qui-quadrado e cálculos de *odds ratio.* O significado estatístico foi fixada em p<0,05.

**Resultados:**

Dos 204 pacientes com fraturas orbitárias incluídos neste estudo, 154 (75,5%) eram sexo masculino (75,5%). A média de idade foi de 42,1 anos. As fraturas orbitárias envolvendo uma parede orbital (58,8%) foram mais comuns do que as que acometeram várias paredes (41,2%). A maioria das fraturas acometeu a parede inferior (60,3%), sendo as paredes mediais as próximas mais frequentemente afetadas (19,6%). A causda mais comum de lesão foi agressão (59,3%), e a segunda mais comum foi queda (24%). A *commotio retinae* foi observada em 20,1% dos casos de fratura orbital e foi mais associada a lesões causadas por agressão (OR=5,22, p<0,001) e menos associada com aquelas causadas por quedas (OR=0,06, p<0,001). As restrições de movimentos oculares eram mais comuns na comoção central do que na periférica (OR=3,79, p=0,015) e com fraturas da parede medial do que com fraturas de outras paredes orbitais (OR=7,16, p<0,001). As chances de comoção não foram maiores em pacientes com fraturas orbitais de paredes múltiplas do que naqueles com fraturas de parede simples (p=0,967).

**Conclusões:**

Na população do estudo, a agressão foi a causa mais comum de fraturas orbitais e resultou em *commotio retinae* mais grave do que qualquer outra causa. Os oftalmologistas devem estar cientes da probabilidade de *commotio retinae* em pacientes com fraturas orbitais resultantes de agressão, independentemente da extensão das lesões do paciente.

## INTRODUCTION

Most eye-related hospital admissions are trauma-induced and 90% are considered preventable^(1,2)^. There have been many epidemiological studies of eye trauma worldwide, however; few have investigated the Bronx population^(3,4)^. The United States Eye Injury Registry (USEIR)^(1)^ was developed to address the deficiency in national data on ocular trauma. Since its inception, it has documented 16,000 serious eye injuries. In 2013, Prevent Blindness America reviewed CDC-funded research and estimated the economic burden of visual impairment in the US to be $139 billion^(2)^. This included the cost of acute and long-term healthcare, government-assisted programs, visual aids, and loss of productivity, both of the patient and their designated caregiver. The decline in quality of life is more difficult to quantify.

A standardized system of classification has been developed that is endorsed by major ophthalmic societies and bodies and the *International Classification of Diseases-Clinical Modifications* (ICD-CM) diagnostic manual. This provides us with a common language and the capacity to compare populations^(5)^. While several international studies have revealed a preponderance of young males among those with ocular injuries, the causes found by different studies are more varied and reflect the environment inhabited by a given population. Scruggs et al. evaluated national trends in ocular injuries using data from the American College of Surgeons National Trauma Data Bank-National Sample Program (2003-2007) and noted a steady increase in ocular trauma over the five years studied from 1.97% of all trauma admissions to 6.00%, with young, male, white patients being the most affected. Common mechanisms of injury were motor vehicle accidents and falls, and the most frequent injury types were orbital injuries and adnexal contusions^(6)^.

Increasingly sophisticated surgical and conservative visual rehabilitation techniques are available to address severe ocular injuries. However, prevention of ocular injuries remains the best way to maintain a fully functional visual apparatus. Knowledge of the incidence, common mechanisms, and at-risk groups among specific populations is instrumental to the development of public health measures aimed at reducing avoidable visual disabilities. Orbital injuries are among the most frequent consequences of facial trauma, and the reported incidence of concurrent orbital and ocular injuries has been found to range from 9%-93% in different populations^(7)^. Fortunately, injuries that pose a serious threat to vision are relatively infrequent^(7,8,9)^. Ocular injuries associated with orbital fracture include ocular and adnexal contusions, eyelid lacerations, hyphema, traumatic iritis, ruptured globes, angle recession, lens dislocation, vitreous hemorrhages, choroidal ruptures, commotio retinae, traumatic macular holes, sclopetaria, retinal tears, retinal detachments, muscle entrapment, and optic neuropathies^(9,10,11,12,13)^.

Commotio retinae is typically considered a mild injury that resolves without sequelae. It presents with whitening of the retina resembling edema but usually includes traumatic disruption of the structural integrity of the inner and outer segments (IS/OS) of the photoreceptors and the retinal pigment epithelium^(14,15)^. Commotio retinae occurs in 2.2%-12.9% of patients with orbital wall fractures^(8,9,10,11)^. Central commotio retinae involving the macula, also known as Berlin’s edema, can lead to subsequent degeneration resulting in visual impairment. To our knowledge, only one study to date has evaluated commotio retinae in orbital injuries. This was part of an analysis of the variables associated with orbital fractures^(10)^. In the present study, we sought to evaluate the relationship between the mechanisms of injury, the types of orbital fractures, and their severity with the concurrence of common ocular injuries, with a particular focus on different types of commotio retinae, in a population of patients presenting to emergency departments in the Bronx, New York.

## METHODS

This retrospective study was approved by the institutional review board of Albert Einstein College of Medicine (AECOM) and was conducted in accordance with the tenets of the 2013 version of the Declaration of Helsinki, and the stipulations of the 1996 Health Insurance Portability and Accountability Act (HIPAA). We evaluated the medical records of patients with computed tomography (CT) scan results confirming trauma-induced orbital fractures. Patients were seen between July 2017 and September 2019 in the emergency departments of six hospitals in the Bronx, New York, one of which is a Level 1 trauma center that serves as the AECOM teaching hospital system. Codes from the ninth edition of the ICD-CM diagnostic manual were used to identify patients presenting with orbital fractures and associated ocular injuries in the electronic medical records.

Inclusion criteria were patients who had sustained a trauma-induced orbital fracture that had been confirmed by CT scan and who underwent an emergency room ophthalmic evaluation during the study period with documented dilated fundus exam findings. Exclusion criteria were trauma patients with no discernible ocular or orbital injuries and patients with incomplete records of the required clinical or demographic data. The data tabulated for each patient were demographic information, the circumstances of injury, ophthalmic examination results, CT scan findings, and whether admission was required. Snellen visual acuity test results were converted to a logarithm of the minimum angle of resolution (log Mar) to facilitate numerical analysis. Clinical and demographic variables were compared using two-tailed student’s t-tests, chi-squared tests, and odds ratio calculations. These were performed on Stata v. 14.2 (StataCorp., College Station, Texas, USA) software. Additional sub-analyses that stratified the population by age and sex were also performed. Central (Berlin’s edema) and peripheral commotio were combined in the initial analysis and then divided for the subsequent subanalysis and the analysis of correlations with demographic variables, injury mechanisms, and orbital trauma variables. Statistical significance was set at p<0.05.

## RESULTS

**Figure 1:**
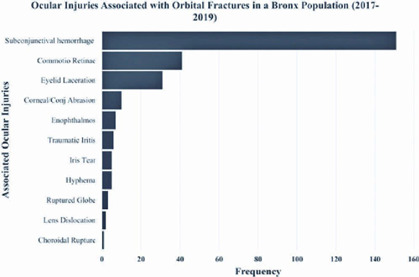
Ocular injuries associated with orbital fractures. Subconjunctival hemorrhages, commotio retinae, and lacerations were the ocular injuries most frequently associated with orbital fractures.

**Figure 2:**
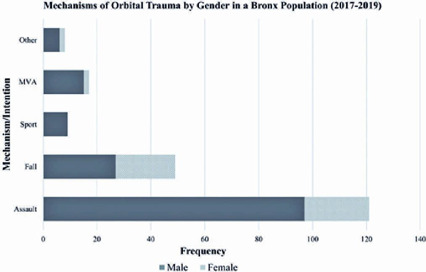
Mechanisms of orbital fractures in a population of the Bronx, New York.

We identified two hundred and nineteen orbital trauma patients in the medical records, of whom, 204 met our inclusion criteria (Table 1). The mean (SD) age was 42.1(20.1) years (median 37; IQR=26-55). The majority of the patients were male (75.5%). The most frequently affected orbital wall was the orbital floor (60.3%), followed by the medial wall (19.6%), the orbital roof (9.3%), and the lateral wall (4.9%). Zygomatic complex fractures made up 5.8% of our cases. Most patients had fractures to one (58.8%) or two (32.4%) orbital walls. Ocular injuries that were frequently associated with orbital fractures were subconjunctival hemorrhages (51.5%), commotio retinae (20.1%), eyelid lacerations (15.2%), and corneal/conjunctival abrasions (4.9%) (Figure 1). Most patients had the full range of eye movements (47.6%) or restricted eye movements (40.2%), with only a few recorded as spontaneous, unable, or unknown. Overall, assault was the most common mechanism of injury, making up nearly 60% of all cases. Falls were the second most common, causing 24% of injuries. Motor vehicle accidents and sports injuries were less frequent, comprising 8.3% and 3.9% of injuries, respectively (Figure 2). Other causes included firearms and work-related injuries. The associations of commotio retinae with diffe rent causes of orbital fractures are outlined in Table 2.

**Table 1:** Demographic and clinical characteristics of patients who sufered trauma-induced orbital fractures in the Bronx, New York between July 2017 and September 2019

Characteristics	Number	Percentage (%)	Characteristic	Number	Percentage (%)	Mean (SD)	Median (IQR)
Gender			**Age (years)**			42,1 (20.1)	37 (26-55)
Male	50	24.5	0-9	4	2.0		
Female	154	75.5	10-19	19	9.3		
			20-29	40	19.6		
			30-39	46	22.6		
Mechanism of injury			40-49	22	10.8		
Assault	121	59.3	50-59	35	17.2		
Fall	49	24.0	60-69	15	7.4		
Motor vehicle accident	17	8.3	≥70	23	11.3		
Sport-related	9	4.4					
Other	8	3.9					
			**Visual acuities**				
			Log Mar OD	131		0.1 (0.2)	0 (0-0.1)
			Log Mar OS	130		0.2 (0.2)	0.1 (0-0.3)
Orbital wall(s) involved			**Extraocular muscle restrictions**				
Inferior	123	60.3	Full	97	47.6		
Lateral	10	4.9	Restricted	82	40.2		
Medial	40	19.6	Spontaneous	6	2.9		
Superior	19	9.3	Unable	15	7.4		
Zygomatic	12	65.9	Unknown	4	2.0		
Number of walls affected			**Commotio retinae**				
1	120	58.8	Total commotio	41	100		
2	66	32.4	Central commotio	19	46.3		
3	16	7.8	Peripheral commotio	33	80.5		
4	2	1.0	Combined central/peripheral	11	26.8		
Associated ocular injury			**Commotio by gender**				
Sub-conjunctival hemorrhage	105	51.5	Male	36	87.8		
Laceration	31	15.2	Female	5	12.2		
Corneal/conjunctival abrasions	10	4.9					
Enophthalmos	7	3.4	**Commotio by age (years)**				
Commotio retinae	41	20.1	<21	6	14.6		
Trauma iritis	6	2.9	21-64	35	85.4		
Hyphema	5	2.5	≥65	0	0		
Lens dislocation	2	1.0					
Ruptured globe	3	1.5	**Comparative LogMar visual acuities of affected eyes**				
Iris sphincter tear	5	2.5	Central commotio (alone)	11		0.27 (0.32)	0.1 (0-0.04)
Choroidal rupture	1	0.5	All other injuries	114		0.16 (0.24)	0.1 (0-0.3)

SD= standard deviation; IQR= interquartile range.

**Table 2:** Association of commotio retinae with different types and causes of traumatic orbital wall fractures in a Bronx population (2017-2019)

Mechanism	Total	Frequency	Percentage	Odds ratio	Confidence interval	p-value
**Assault**	**121**	**35**	**28.9**	**5.22**	**2.01–15.91**	**0.0001**
**Falls**	**49**	**1**	**2.0**	**0.06**	**0.001–0.38**	**0.0003**
Sports-related	9	1	11.1	0.48	0.01–3.81	0.4914
MVA	17	3	17.7	0.84	0.15–3.24	0.7922
Others	8	1	12.5	0.56	0.01–4.55	0.5843
**Orbital wall fractured**						
Medial	95	24	25.3	1.83	0.87–3.92	0.086
Lateral	51	12	23.5	1.32	0.56–2.97	0.480
Floor	143	27	18.9	0.78	0.36–1.76	0.507
Roof	20	3	15.0	0.68	0.12–2.53	0.549

MVA = motor vehicle accident.

Males outnumbered females for all mechanisms of orbital injury. Assault was the most common cause in males (OR=1.84, 95%CI=0.92-3.69; p=0.061) and in patients in the 21-64 years age group (OR=4.99, 95%CI=2.48-10.15; p<0.001). Falls were the most common cause in females (OR=3.69, 95%CI=1.73-7.83; p<0.001) and in those aged ≥65 years (OR=28.07, 95%CI=9.71-90.28; p<0.001). Patients aged <21 years group were most likely to have sports-related injuries (OR=9.83, 95%CI=1.92-52.39; p<0.001). Commotio was observed more frequently in males than females (OR=2.75, 95%CI=0.92-9.49; p=0.040) and those aged 21-64 more often than in any other age group (OR=2.81, 95%CI=1.07-8.65; p=0.024).

**Figure 3:**
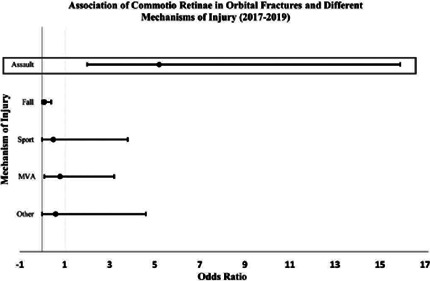
Associations between commotio retinae and various mechanisms of orbital fracture injury.

**Legend**: Summary of a logistic regression analysis of the associations between commotio retinae and the different mechanisms of orbital fractures. Assault injuries were the most commonly associated with commotio retinae. MVA = motor vehicle accident.

Concerning types of orbital fractures, roof fractures were most associated with falls (OR=3.72, 95% CI=1.28-10.67; p=0.004) but fractures to other orbital walls did not exhibit strong associations with specific mechanisms of injury. Combined peripheral and central commotio (Berlin’s edema) was most likely to occur after assault injuries (OR=5.22; 95%CI=2.01-15.91; p<0.001), and least likely after falls (OR=0.60; 95%CI=0-0.001-0.38; p<0.001) (Figure 3). Although commotio was most frequently associated with medial wall fractures (OR=1.83; 95%CI=0.87-3.92; p=0.086), the correlation was not statistically significant. More extensive orbital fractures, defined as the involvement of >1 orbital wall, did not predict a higher chance of commotio (OR=1.01, 95% CI=0.47-2.14; p=0.967) than single wall fractures.

When commotio was separated into central and peripheral commotio, the two forms yielded similar results, with both predominantly associated with assault injuries, (OR=4.06; 95%CI=1.10-22.37; p=0.020 and OR=6.23; 95%CI=2.04-25.24; p<0.001, respectively) (Table 3). However, while no association was found between peripheral commotio and any specific orbital wall, central commotio was most often associated with medial wall fractures (OR=7.16; 95%CI=1.94-39.28; p<0.001) (Table 4). Cases of central commotio also showed a greater likelihood of eye movement restrictions than peripheral commotio (OR=3.79, 95%CI=1.14-16.19; p=0.015) (Table 5). The visual acuity, measured by Snellen eye tests, of patients with central commotio (log Mar 0.27; Snellen ~20/37) did not differ significantly from that of patients with other ocular injuries (log Mar 0.16; Snellen ~20/28) (p=0.145). No other ocular injury type showed a significant association with specific wall fractures or eye movement restriction.

**Table 3:** Association of commotio retinae with different types and causes of traumatic orbital wall fractures in a Bronx population (2017-2019)

Mechanism	Total	Frequency	Percentage	Odds Ratio	Confidence Intervals	p-value
**Central commotio**						
**Assault**	**121**	**16**	**13.2**	**4.06**	**1.10–22.37**	**0.020**
**Falls**	**49**	**0**	**0**	**0.00**	**0.00–0.57**	**0.010**
Sports-related	9	0	0	0.00	0.00–4.12	0.325
MVA	17	2	11.8	1.33	0.14–6.55	0.717
Other	8	1	12.5	1.41	0.03–2.04	0.752
**Peripheral commotio**						
**Assault**	**121**	**29**	**24.0**	**6.23**	**2.04–25.24**	**<0.001**
**Falls**	**49**	**1**	**2.0**	**0.080**	**0.00–0.51**	**0.002**
Sports-related	9	1	11.1	0.637	0.01–5.04	0.673
MVA	17	1	5.9	0.303	0.01–2.10	0.229
Other	8	1	12.5	0.732	0.02–6.03	0.773

MVA= motor vehicle accident.

**Table 4:** Association of commotio retinae with the type of orbital wall fracture in a Bronx population (2017-2019)

Wall	Total	Frequency	Percentage	Odds Ratio	Confidence Intervals	p-value
**Central commotio**						
**Medial**	**95**	**16**	**16.8**	**7.16**	**1.94–39.28**	**<0.001**
Lateral	51	5	9.8	1.08	0.29–3.39	0.890
Floor	143	12	8.4	0.71	0.24–2.24	0.488
Roof	20	1	5.0	0.49	0.01–3.45	0.485
**Peripheral commotio**						
Medial	95	17	17.9	1.27	0.56–2.87	0.534
Lateral	51	10	19.6	1.38	0.54–3.31	0.442
Floor	143	22	15.4	0.83	0.35–2.04	0.638
Roof	20	2	10.0	0.55	0.06–2.49	0.430

**Table 5:** Association of commotio retinae and extraocular muscle movements in traumatic orbital wall fractures in a Bronx population (2017-2019)

EOM Movements	Total	Frequency	Percentage	Odds Ratio	Confidence Intervals	p-value
**All commotio**						
Full EOM	97	15	15.5	0.57	0.26-1.22	0.116
Restricted EOM	107	26	24.3	1.76	0.82-3.83	0.116
**Central commotio**							
**Full EOM**	**97**	**4**	**4.1**	**0.26**	**0.06-0.88**	**0.015**
**Restricted EOM**	**107**	**15**	**14.0**	**3.79**	**1.14-16.19**	**0.015**
**Peripheral commotio**						
Full EOM	97	14	14.4	0.78	0.34-1.77	0.520
Restricted EOM	107	19	17.8	1.28	0.57-2.95	0.520

EOM= extraocular muscles

Bar chart showing the distribution of mechanisms/causes of traumatic orbital injuries. Assault was the most frequent cause, and, in all mechanisms, males outnumbered females.

## DISCUSSION

In this study, we set out to assess the mechanisms and degrees of orbital fracture injuries and their relationship to concurrent ocular trauma, with special attention to commotio retinae, in patients presenting to emergency departments in the Bronx, New York. We found that the orbital floor and medial wall were the most frequently involved orbital walls in traumatic ocular injuries. Overall, assault was the most common mechanism of injury. However, the frequency of different mechanisms of injury varied between age groups. Patients younger than 21 years were most likely to suffer sports-related injuries, while patients aged 21-64 years most frequently sustained orbital fractures following an assault, and those over 65 years were most likely to have fallen. Males were more likely to be assaulted than females while females were more likely to incur their injury by falling than males.

Demographic differences in mechanisms in injury and the predominance of floor and medial wall fractures affirmed the findings of previous studies of orbital fractures^(10,11,12,13,16,17,18)^. Variations in the mechanisms of injury between different studies most likely reflect the different populations studied. Zagelbaum et al. also conducted a study of ocular trauma in the Bronx and, like us, found a high assault rate. This was 28% in their study, ranking second behind struck by or against an object^(3)^. In their study of orbital fractures at a metropolitan Level 1 trauma center in Chicago, Chiang et al. found assault to be the most frequent mechanism of injury, with a rate of 38.6%^(18)^. However, the rates in the latter two studies were much lower than the rate in the present study, which was close to 60%. This disparity is likely due to our focus on orbital injuries rather than all forms of ocular trauma^(3)^ and the use of different datasets. While the two studies above were both conducted in a single center^(3,18)^, our study combined data from private and public hospitals, which were contained within one system of electronic medical records. We suspect that the higher assault rate found in our population resulted from the inclusion of public hospital data and the rate of violent crime in the Bronx, which was the highest of all New York City boroughs during the period studied^(19)^.

We found subconjunctival hemorrhage, commotio retinae, and eyelid lacerations to be the most common forms of concurrent ocular trauma in patients with orbital fracture. However, only commotio retinae was associated with mechanisms of injury, type of orbital trauma, and restricted eye movement due to extraocular muscle entrapment. Assault injuries were most likely to result in commotio while injuries resulting from falls were least likely. Consequently, males and patients in the 21-64 years groups were more likely to have commotio than other demographic groups with lower rates of assault. Interestingly, more extensive orbital fractures, defined as those involving more than one orbital wall did not increase the odds of commotio.

In a recent study at a military medical center, Blegen et al. evaluated the concurrence of commotio retinae in patients with orbital fracture and found commotio in 12.9% compared to a rate of 20% in our study^(10)^. Like us, they found that commotio was more often associated with assault injuries (60.5%) than falls (24.1%). Most cases occurred in those with fractures to the inferior orbital wall (47.4%) and the inferior portion of the retina was the most frequently affected. Of clinical consequence, they found that these patients were more likely to suffer muscle entrapment and require surgery. Our study found a greater propensity for commotio in medial orbital wall fractures, but not to a statistically significant level (p=0.086). However, when we evaluated central (Berlin’s edema) and peripheral commotio separately, we noted that 45.2% of commotio cases were central (macular) and these cases exhibited strong associations with medial wall fractures (p<0.001) and extraocular muscle entrapment (p=0.015).

Disparities between the Blegen et al. study and our own likely resulted from differences in the study design and the different rates of injury causes observed. Although Blegen et al.’s study found a similarly strong association between assault and all commotio to ours, the incidence of assault injuries (39.5%) was lower than ours (60%). This lower incidence of assault injuries probably accounts for the lower rate of macular commotio (22.2%). These findings suggest that the mechanism of injury has a greater effect on the photoreceptor disruption that manifests as commotio than does the extent of the injury. We posit that, when the trauma is caused by assault, there is direct force applied to the globe and this is less often the case with other causes of orbital fractures. Therefore, a higher index of suspicion for commotio is warranted when assessing assaulted patients with orbital fracture. We found a strong correlation between central commotio retinae and medial wall fracture. Similarly, Smith et al. found that, in patients with orbital fractures, retinal pathology was most likely to occur in patients with medial wall fractures^(20)^. This may be because the angle and force involved in these traumatic injuries result in a greater impact on the globe. However, few studies have focused on commotio in orbital fractures and additional research is necessary to confirm this association and identify the cause.

Several studies have developed predictive models to help identify those patients with orbital fractures at greater risk of the concurrence of severe ocular trauma requiring immediate ophthalmic assessment or inter-vention^(7,8,9,21)^. The sensitivities and specificities of these models differ due to variability in the methodologies employed and the definitions of injury severity. In a recent report, Rossin et al. defined substantial ocular trauma as retrobulbar hemorrhage, hyphema, ruptured globe, large corneal abrasion, and intraocular pressure greater than 30 mmHg. They determined that blunt trauma with a foreign object, visual acuity insufficient to count fingers, diplopia in the primary position, conjunctival contusion, and orbital roof fractures were most predictive of the concurrence of substantial ocular trauma. Their ranking of ocular injuries defined commotio retinae as insubstantial, requiring only outpatient follow-up^(9)^. Interestingly, the rate of commotio found in their study was only 7.4%. Although our analysis did not reveal significant differences in visual acuity between patients with central commotio and other ocular injuries based on the emergency department assessments, we observed an association between central commotio and both direct blunt trauma (assault) and restricted eye muscle movement. However, Rossin’s team defined these signs as features of orbital injury likely to be associated with substantial ocular injury.

There have been few reports on the sequelae of commotio and those that exist suggest that commotio may be followed by anything from complete recovery^(10,22,23,24)^ to the subsequent development of cystic or pigmentary macular degeneration, macular holes, retinal tears, or retinal detachment, resulting in an irreversible impairment of visual function^(22,23,24,25,26,27,28)^. Recent optical coherence tomography (OCT) evaluations of central commotio have identified disruption between the inner and outer photoreactor segments (in the ellipsoid zone), intraretinal hyperreflectivity, microvascular disruption, and disorganization and atrophy of the inner retinal and outer nuclear layers^(14,25,26,27,28,29,30,31)^. Chen et al. used spectraldomain OCT to grade macular commotio and found both foveal thickness and the degree of outer retinal atrophy to be significantly correlated with visual acuity after six months. Indeed, OCT angiographic studies have detected visual deterioration even in patients with good vision at presentation^(26)^. Thus, we recommend ophthalmic examinations of all patients presenting with orbital fractures and regular follow-ups with dilated fundoscopy and ancillary OCT evaluations in those with commotio retinae.

This study had some limitations. These include the retrospective study design, which meant that the data collected was limited to that recorded by emergency room staff, which did not detail the specifics of each assault or the number of impacts to the eye. Also, the limited population of patients presenting to a subset of hospitals in one New York City hospital system may limit the generalizability of our results to a broader population. The study only included the initial examination conducted by on-call residents in the emergency department, so details on the follow-up periods were unavailable. This prevented any evaluation of ultimate visual recovery or long-term impairment resulting from the injuries sustained. Furthermore, our analysis of vision determined the best-corrected pinhole vision of patients, not refractive vision, and did not consider previous visual acuity. The effects of injuries on vision at presentation might be a better indicator of patient prognosis. However, this was not logistically feasible with this study design. Despite these limitations, this study affirmed that commotio retinae is a common occurrence following orbital fractures and is strongly associated with blunt ocular trauma from assault injuries. Interestingly, central commotio retinae was related to medial wall fractures but not to the extent of the injury, as measured by the number of walls fractured. This has implications for management, indicating the advisability of ophthalmic assessments, including dilated fundoscopy, for all patients with orbital fractures, regardless of the degree of injury. Future studies that include a broader population will have larger samples that are better powered to analyze the relative associations of other ocular injuries, including ruptured globes and choroidal ruptures, of which the present study found only three and one cases, respectively. Furthermore, OCT evaluations and long-term follow-up would augment our current findings and provide information about the outcomes and complications of commotio retinae.

In the population studied, assault was the most common cause of orbital fractures and was more frequently associated with the concurrence of commotio retinae than other mechanisms of injury. Medial wall fractures and limited extraocular muscle movements were significantly correlated with central commotio. However, the extent of the orbital injury, as measured by the number of walls fractured, was not associated with either central or peripheral commotio. Ophthalmologists should have a high index of suspicion for commotio retinae in patients presenting with orbital fractures resulting from direct blunt trauma.
